# Evaluation of the impact of the voucher and accreditation approach on improving reproductive behaviors and status in Cambodia

**DOI:** 10.1186/1471-2458-11-667

**Published:** 2011-08-24

**Authors:** Benjamin Bellows, Charlotte Warren, Saphonn Vonthanak, Chhea Chhorvann, Hean Sokhom, Chean Men, Ashish Bajracharya, Ubaidur Rob, Tung Rathavy

**Affiliations:** 1 Population Council, General Accident House, Ralph Bunche Road, PO Box 17643-00500, Nairobi, Kenya; 2 National Institute of Public Health, No.2, Kim Yl Sung Blvd, Khan Toul Kork, Phnom Penh, Cambodia; 3 Research Unit, National Center for HIV/AIDS, Dermatology and STDs (NCHADS), Phnom Penh, Cambodia; 4 Center for Advanced Studies (CAS), #160, Street 156, Sangkat Teuk Laak 2, Tuol Kork, Phnom Penh, Cambodia; 5 Population Council, Vietnam, Unit 17-04 Prime Center, 53 Quang Trung Street, Hai Ba, Trung District, Hanoi, Vietnam; 6 Population Council, Bangladesh, House 21, Road 118, Gulshan, Dhaka 1212, Bangladesh; 7 Ministry of Health, National MCH Center (NMCHC), Rm 318, 2nd Fl. National MCH Institute bldg. #31A Street 47 (Street France) Sangkat Srah Chak, Khan Daun Penh, Phnom Penh, Cambodia

**Keywords:** Vouchers, Output based approach

## Abstract

**Background:**

Cost of delivering reproductive health services to low income populations will always require total or partial subsidization by government and/or development partners. Broadly termed "demand-side financing" or "output-based aid", these strategies include a range of interventions that channel government or donor subsidies to the user rather than the service provider. Initial pilot assessments of reproductive health voucher programs suggest that they can increase access, reduce inequities, and enhance program efficiency and service quality. However, there is a paucity of evidence describing how these programs function in different settings for various reproductive health services.

**Methods/Design:**

Population Council, funded by the Bill and Melinda Gates Foundation, intends to generate evidence around the "voucher and accreditation" approaches to improving the reproductive health of low-income women in Cambodia. The study comprises of four populations: facilities, providers, women of reproductive age using facilities, and women and men who have been pregnant and/or used family planning within the previous 12 months. The study will be carried out in a sample of 20 health facilities that are accredited to provide maternal and newborn health and family planning services to women holding vouchers from operational districts in three provinces: Kampong Thom, Kampot and Prey Veng and a matched sample of non-accredited facilities in three other provinces. Health facility assessments will be conducted at baseline and endline to track temporal changes in quality-of-care, client out-of-pocket costs, and utilization. Facility inventories, structured observations, and client exit interviews will be used to collect comparable data across facilities. Health providers will also be interviewed and observed providing care. A population survey of about 3000 respondents will also be conducted in areas where vouchers are distributed and similar non-voucher locations.

**Discussion:**

A quasi-experimental study will investigate the impact of the voucher approach on improving reproductive health behaviors, reproductive health status and reducing inequities at the population level and assess effects on access, equity and quality of care at the facility level. If the voucher scheme in Cambodia is found effective, it may help other countries adopt this approach for improving utilization and access to reproductive health and family planning services.

## Background

Stagnating indicators for several reproductive and child health conditions in many countries of Africa and Asia are a major concern for national governments and development partners striving to achieve the Millennium Development Goals (MDGs). Universally, these indicators, and especially maternal and infant morbidity and mortality, are poorest among low-income populations. Weak and inefficient health systems sustain these inequities in access to and use of essential health services. There remains an over-reliance on financing the inputs of service delivery in the public sector, supported by the beliefs that: (i) service purchasing is too difficult, and (ii) that the private sector is not willing or able to serve low income clients.

Recognizing that the cost of delivering reproductive health services to low-income populations will always require total or partial subsidization by the government and/or development partners, alternatives to the traditional 'supply-side' approach to financing service delivery are being explored. Broadly termed "demand-side-financing" (DSF) or "output-based aid" (OBA), these alternatives include a range of interventions that channel government or donor subsidies to the service user rather than the service provider [1,2]. The goal is to increase access to and use of key services by subsidizing the user with sufficient resources to enable them to purchase the required service and to choose a provider from a number of alternatives. Doing this stimulates a market for the services, which creates competition between providers thereby motivating improvements in access to and quality of services to be able to attract users financially empowered with the means to pay. Providers that perform well by attracting and serving users are thus rewarded by receiving payments for their services that both fully cover the service delivery costs and provide some additional funds as an incentive.

An OBA approach, therefore, uses explicit performance-based subsidies to motivate providers to deliver selected reproductive health services at a specified level of quality and at an affordable cost so that the economically disadvantaged are not excluded. Several interventions are currently being developed and tested, including franchising and contracting, social health insurance, conditional cash transfers and vouchers. Social insurance programs are intended to achieve a number of policy goals: i) by reducing financial barriers, they can increase access to services generally, and reduce inequities by making them affordable to the poor and other underserved groups; ii) by accrediting many providers to offer the service at the same price, they can increase choice for clients; iii) by including more than one provider, competition for member clients can increase efficiency in delivery and possibly reduce prices further; and iv) by requiring a minimal level of quality to be accredited, quality of care can be improved.

OBA for reproductive health are not new; Taiwan and Korea successfully used the strategy in the 1960s to increase access to family planning [3], and Nicaragua implemented two OBA projects for sexually transmitted infection (STI) services in 1995 [4]. A study from India showed that an OBA voucher scheme could be an option for increasing the utilization of reproductive and child health services [5]. In the Yunnan Province of China, an output-based voucher scheme was introduced for low-income, pregnant women to enhance the utilization of maternal and child health services. This scheme covered the cost of antenatal care (ANC), delivery and postnatal care (PNC) as well as care of sick children. Findings show that the OBA program increased the utilization of treatment for childhood diarrhea among the poor [6]. More recently there has been a significant increase in interest in OBA. Key development partners interested in voucher schemes for reproductive health include the World Bank, Canadian International Development Agency (CIDA), Department for International Development (DFID) and the German Government. Specifically, the German Development Bank (KfW) is currently supporting OBA programs for reproductive health in collaboration with government and non-government partners in Kenya, Uganda, Tanzania and Cambodia. Between them, these programs are or will pilot-test OBA schemes for deliveries assisted by skilled personnel, family planning, prevention and management of STIs, and care for survivors of sexual assault.

In brief, these programs generally establish a management agency that sells subsidized vouchers to clients, who purchase a voucher for a specific service and usually at a price that has been determined to be affordable for the lowest-income clients. The program also identifies and invites a number of service providers (individually or within an organization, which can be public, non-profit or for-profit) to participate in the program. Those agreeing to participate can only do so if they can demonstrate being able to provide the service(s) at a specified standard of care; once this is demonstrated, they are then accredited to participate subject to their accreditation being regularly reviewed. When the client needs the services, s/he then redeems the voucher for the specified service at one of the accredited providers/facilities. The service provider is then reimbursed the full cost of providing the service upon submission of the voucher and supporting evidence to the VMA.

Initial findings from the few assessments of reproductive health V&A programs suggest that, if implemented well, they have great potential for achieving the policy objectives of increasing access and use, reducing inequities and enhancing program efficiency and service quality [7-9]. At this point in time, however, there is a paucity of evidence describing how the various V&A programs function in different settings, for various RH services and for services delivered through public, for-profit or non-profit organizations and how the V&A program affects the operational efficiency and business model used by service delivery organizations and individual providers. There is also limited understanding of their effect on the quality of care received by clients and on levels of service utilization, especially among the poor and underserved. And most importantly, there is no evidence to date on their impact on RH behaviors and status at the individual and population levels, especially on those health status indicators relevant for the MDGs.

### Cambodia- Output Based Approach (OBA) to Social Health Insurance

In 2010 the Cambodia Ministry of Health, with technical support from a consortium of EPOS Health Consultants, Oxford Policy Management (OPM), PriceWaterhouseCoopers (PwC), and Action for Health (AfH) Cambodia, developed a pilot reproductive health voucher scheme offering three types of vouchers (family planning, maternal care, and abortion services). The overall goal of the voucher pilot is to contribute toward the MDG target of lower maternal mortality. The purpose of the pilot is to test whether the combination of provider reimbursements and quality reviews will increase utilization of quality maternal services by poor women. The first phase of the voucher project will contract approximately 110 health centers in three operational districts of Kampong Thom province (Kampong Thom, Staung and Baray Santuk), three operational districts of Kampot province (Kampong Tralach, Angkor Chey and Chhouk) and three operational districts of Prey Veng province (Pearaing, Preah Sdach and Kampong Trabek). The voucher transfers purchasing power to the poor who choose which facility they will visit and the providers are reimbursed for their services from the VMA. Clients may purchase three types of vouchers: family planning, maternal health services, and abortion services.

## Methods/Design

### Hypotheses to be tested

#### (i) At facility level

a) Participating facilities will have a greater increase in average utilization of voucher subsidized reproductive health services compared to control facilities between baseline and endline surveys.

b) Participating facilities will have a greater increase in the proportion of poor clients for essential maternal health services compared to control facilities between baseline and endline surveys.

c) The quality of voucher subsidized reproductive health services in voucher facilities will be equal to or greater than the quality of the same in non-voucher facilities.

#### (ii) At population level

a) Communities near to voucher facilities offering maternal health services will have greater increase in the proportion of family planning visits, abortion consultations, ANC visits, safe delivery at facility, and PNC visits by trained providers among the poor compared to communities far from voucher facilities in the endline survey.

### Study Objectives

This protocol describes an evaluation of the impact and effectiveness of the maternity and abortion services voucher program in Cambodia. The study has two specific aims:

1. Assess the effect of the voucher and accreditation (V&A) approach on increasing access to, quality of, and reducing inequities in the use of selected RH services:

2. Evaluate the impact of the voucher and accreditation (V&A) approach on improving RH behaviors and status and reducing the inequities at the population level.

### Study Design

A quasi-experimental design will be followed in which the health facility assessments and population surveys are undertaken among the target populations before and after the voucher's introduction and also among an equivalent comparison population living in areas not exposed to the voucher in order to control for potential time dependent confounding among observed variables.

Operational districts (OD) are the primary sampling unit. The nine ODs targeted for vouchers are matched with nine control ODs. The list of 18 potential ODs (9 voucher ODs and 9 controls) is used to draw a logistically feasible series of OD pairs matched on HEF status - when HEF information is available. The second stage will then purposively select 20 voucher and 20 control facilities. The list of OD pairs are listed in Table 1.

**Table 1:** List of OD pairs selected based on presence of specific healthcare finance strategies (data from URC-CHS Cambodia office)

OD pairs	Operational District(s)	Province	Control Voucher	Donor	Implementer	Operator	Start date	Total population
1	Kampong Thom	Kampong Thom	Voucher	HSSP/WB	AFH	AFH	Oct-05	264,140
1	Kampong Cham-Kampong Siem	Kampong Cham	Control	BTC	BTC	AFH	Sep-05	129,860
2	Stong	Kampong Thom	Voucher	HSSP/WB	AFH	AFH	Oct-07	140,733
2	Kralanh	Siem Reap	Control	BTC	BTC	CHHRA	Apr-06	136,520
3	Preah Sdach	Prey Veng	Voucher	HSSP/ADB	URC-CHS	AFH	Sep-08	114,788
3	Samrong	Oddor Mean Chey	Control	BTC	BTC	CHHRA	Jan-05	105,488
4	Pearaing	Prey Veng	Voucher	HSSP/ADB	URC-CHS	AFH	Jul-02	188,230
4	Svay Rieng	Svay Reing	Control	UNICEF	UNICEF	HFSC	Jul-02	311,473
5	Kampong Trach	Kampot	Voucher	Government	ODO	ODO	Jan-08	163,362
5	Angkor Chey	Kampot	Control	Government	ODO	ODO	Jan-08	117,325
6	Kampong Trabek	Prey Veng	Voucher	Government	ODO	ODO	Jan-08	134,163
6	Romeas Hek	Svay Reing	Control	Government	ODO	ODO	Jan-08	137,056
7	Chhouk	Kampot	Voucher					189,566
7	Kepville	Kep	Control					33,306
8	Baray Santuk	Kompong Thom	Voucher					243,435
8	O Reang Ov - Kaoh Soutin	Kampong Cham	Control					103,258
9	Prey Veng	Prey Veng	Voucher					212,689
9	Neak Loeung	Prey Veng	Control					185,283

#### Facilities

Facilities are the secondary sampling unit and voucher facilities will be drawn from the pilot regions of Kampong Thom, Kampot, and Prey Veng; non-voucher facilities will come from nearby control ODs. Voucher and control facilities will be propensity score matched by ownership type (government and private) and by level of obstetric care (basic or comprehensive) to maximize the likelihood of patients having similar social, cultural, economic characteristics, and RH behaviors.

#### Service providers

The knowledge and experiences of service providers in both voucher and non-voucher facilities will be assessed. All eligible service providers available on the day of data collection at the facility will be interviewed to measure indicators concerning provision of maternal and neo-natal health (MNH), family planning and abortion services. Service providers responsible for maternal health and abortion services within the facility will be eligible for inclusion.

#### Key informants

The study will also carry out in-depth interviews (IDIs) with these key informants at the VMA, NGOs managing health equity funds (HEFs), health facilities, government and other stakeholders. These interviews will be used to gain a deeper understanding into the motivations, perceptions, and priorities of the stakeholders.

#### CPI surveys of quality of care for reproductive health care consultations

Measurement of the quality of family planning, abortion and maternity services will be conducted at designated consultation times. Samples of clients attending each type of consultation will be recruited if they meet the following eligibility criteria:

• Are accessing maternity care including postnatal care, for themselves (and/or their newborns), at delivery or one of the pre-discharge, six-week postpartum consultation times;

• Are aged 18 years or older (the small proportion of clients that are less than 18 years will not justify the difficulties in obtaining parental/guardian permission for legal minors);

• Are aged below 46 years (the small proportion of women giving birth above this age will be excluded);

• Give their informed consent for their consultation to be observed and the key actions taken recorded, and to be interviewed on exiting from the consultation.

All women satisfying these inclusion criteria will be recruited until the required sample sizes have been reached.

#### Client exit interviews

Client exit interviews will be conducted to measure the quality and satisfaction level of family planning, abortion and maternity services immediately following the consultation with service providers. Samples of clients attending each type of consultation will be recruited if they meet the following eligibility criteria:

• Are accessing family planning, abortion, or essential maternity care including ANC, PNC, for themselves (and/or their babies), at pre-discharge following delivery and six-week postpartum consultation times;

• Are aged 18 years or older;

• Are aged below 46 years;

• Provide consent for exit interview.

All women satisfying these inclusion criteria will be recruited until the required sample sizes have been reached.

#### Population survey respondents

Baseline and endline surveys will take place in the period between early 2011 and mid-2012 to compare patterns of service use and perception and to compare any differences between communities that have ready access to the Voucher and communities that do not have access. Each fieldworker has a catchment area and will visit households regularly for collecting the list of pregnant women. These lists will be used as sampling frame to draw required number of sample through simple random sampling procedure. Respondents will be interviewed if they meet the following eligibility criteria:

• Used family planning or delivered in the last one year preceding the survey.

• Are aged over 18 years (the small proportion of clients that are less than 18 years will not justify the difficulties in obtaining parental/guardian permission for legal minors);

• Are aged below 46 years (the small proportion of women giving birth/accessing FP above this age will be excluded);

• Provide consent for interviews.

#### Sample size

In the 2005 DHS, the national proportion of facility-based births was 22% of all births in the previous five years. For purposes of this study, the national figure is considered as baseline level of facility-based births in the voucher region. To detect a 10% increase in the proportion of facility-based births, 1500 experimental subjects and 1500 control subjects are required to be able to reject the null hypothesis that the proportion of facility-based births for experimental and control subjects are equal with probability (power) 0.8. The Type I error probability associated with this test of this null hypothesis is 0.05. An uncorrected chi-squared test of proportions will be used to evaluate this null hypothesis.

#### Selection process of survey respondents

Survey respondents will be drawn from the facility catchment areas in the selected experimental and control sites. Depending on the final number of facilities enrolled, a complete list of enumeration areas will be made and at least three EAs from sampled sub-district units will be selected through probability proportional to size (PPS) where size is the total population or total number of pregnant women.

From each selected village, required number of respondents will be selected from the list of pregnant mothers prepared by the respective fieldworkers. Within each EA, it is estimated that there are approximately 20 deliveries in 12 month period.

### Data Collection Procedures

#### To Assess the effect of the Voucher on increasing access to, quality of, and reducing inequities in the use of, selected RH services

##### a) Undertake health facility assessments: measuring quality of MNH/RH and other services over time

Population Council and NIPH will conduct two health facility assessments (HFAs) of the quality of care provided in voucher and similar but non-voucher facilities. A baseline assessment will be undertaken to determine the comparability of the facilities and to provide baseline measures of the quality of care. To determine the sustainability of the quality of care a endline assessment will be undertaken 12-18 months later after the baseline.

In addition, data collected through routine monitoring of service statistics (see below) will provide further information about the sustainability of the service configurations in terms of client load, services mix, client characteristics etc. Data collection procedures for facility assessments are as follows:

###### i) Facility inventory

An inventory of available resources to learn about the facility infrastructure, staffing numbers and skills mix, services provided, staff training undertaken, availability of equipment, commodities, test kits, stationary (client cards and notes), medications required to provide the services within the intervention will be undertaken. The head of the facility will be approached to assign a nurse/midwife to facilitate the work of researcher. The nurse/midwife will guide the researcher around the facility to observe and record all relevant information on a checklist.

###### ii) Collecting service statistics

Service statistics and HMIS form will be collected throughout the project period from all selected facilities. The study will also review service statistics (related to routine program data) regarding utilization of maternal health care services for a 12 month period prior to the assessment visit. The number of new and continuing clients coming to a clinic for voucher services as well as other health services will be recorded. Monthly trends in the numbers of new and continuing voucher clients will be obtained from facility records. In addition, program implementation cost will be analyzed from the facility for cost analysis.

###### iii) Interviews with service providers

Service providers' interviews will be conducted to determine their knowledge and skills for RH voucher services, as well as understand the organizational setup and description of related activities. Interviews will also ascertain their perceptions of barriers and operational challenges that may influence voucher holders' acceptance of services and the provider's attitudes towards the accreditation process. All providers involved in voucher service delivery will be approached for interview in each health facility. It is estimated that on average 2-3 service providers will be eligible to participate in the study at each facility. This will give around 120 providers for experimental and comparison groups each (3 providers × 40 facilities). The same number of interviews will be carried out in the endline survey to measure the changes likely associated with the program.

###### iv) Observation of client-provider interactions (CPI)

The CPI encompasses both the **process **(how clients are treated and whether they actively participate) and the **content **(what they are told, technical competence, accuracy of information, provision of essential information) of a consultation. After obtaining informed consent from the client, a structured non-participatory observation of the client-provider interaction will be undertaken to determine the quality of ANC and PNC services provided. It's planned that 1260 clients (6 FP consultations + 6 first ANC + 6 other ANC + 6 1^st ^PNC + 6 2^nd ^PNC * 40 facilities+ 6 abortion consultations * 10 facilities) will be observed through CPI at voucher and non-voucher facilities.

To measure the magnitude of changes in the quality of services provided, composite summary scores will be developed for a series of key indicators by aggregating the mean scores of key items being assessed for each individual client-provider interaction being observed. This scoring system will categorize whether an accepted standard of quality has been met or not. For each study group, a mean score will be calculated for each indicator and then a composite summary score will be calculated which will help to make statistical comparisons between experimental and control groups over time. Examples of the types of individual items and key indicators that will be used are given in Table 2.

**Table 2:** Examples of groups of key actions/indicators to make composite scores of quality of care

Quality of:	Observed provider actions:
a. Client - provider rapport (0-7)	Client greeted warmly, Discussed medical conditions, Asked if client understood information, Encouraged client to ask questions, Used client's name, Help in decision-making, Consultation time > 15 minutes
b. ANC counseling	Birth planning, danger signs, physical and laboratory examinations, vitamin A capsule, iron tablet/syrup, TT vaccination, infant feeding, fertility intentions
c. PNC counseling on danger signs since childbirth (0-10)	Ask about: bleeding since birth, color/smell of vaginal discharge, condition of perineum/CS scar, fever, headache or blurred vision, swelling in face, hands or feet, signs of thrombophlebitis, tiredness or breathlessness, convulsions or fits, LAM, breastfeeding

The proportion of women receiving an acceptable quality of service will be calculated in addition to the mean scores. This is because the mean score may be high if a small proportion of clients receive excellent services. The methodology to calculate the proportion of women receiving an acceptable quality is similar to the Lot Quality Assurance Sampling (LQAS) approach that has been used in Kenya and elsewhere for assessing quality [10]. LQAS follows the principle that an entire group (lot) of services is deemed poor quality if a certain proportion within a small sample does not reach a minimum standard. LQAS applies cumulative probabilities calculated with a binomial formula to select small sample sizes and decision criteria for judging a group of providers.

In consultation with the program management, the minimum standard for quality maternal health care services will be reviewed and adapted by building on existing guidelines and protocols and previous studies.

###### v) Client exit interviews

Exit client interviews will be conducted with women aged 18-45 years who receive family planning consultation, abortion consultation, ANC or PNC care services in intervention and control areas. Six clients per service will interviewed per facility for a total of 1260 clients (6 family planning + 6 first ANC + 6 other ANC + 6 1^st ^PNC + 6 2^nd ^PNC clients * 40 facilities + 6 abortion clients * 10 facilities) women will be interviewed. The exit client interviews will provide information about accessibility to services, the clients' attitude towards receiving services from the facility, and quality of services received from the facility with regard to privacy, confidentiality, non-judgmental attitude and respect. To determine the sustainability of the quality of care provided, exit interviews will be undertaken periodically to determine the extent to which the quality of care has changed.

###### vi) In-depth interviews with key informants

Interviews will be conducted with relevant stakeholders to determine their knowledge regarding the voucher as well as understand the organizational setup and operational issues. Interviews will also ascertain their perceptions of barriers and operational challenges that may influence clients' acceptance of services and the provider's attitudes towards the accreditation process. Approximately 50 key informants will be interviewed.

##### Evaluate the impact of the voucher and accreditation (V&A) approach on improving RH behaviors and RH status and reducing the inequities at the population level

###### a) Population based survey

Population Council and the Center for Advanced Studies (CAS) will conduct population-based surveys with a randomly selected sample of women aged 18-45 years from the catchment communities of selected study facilities. The intention of the population-based surveys is to build a view of the women's utilization of services using vouchers at facilities in the region. The second half of the cross sectional survey will be carried out in the catchment area of control facilities. An endline survey will be conducted in the same areas in early to mid-2012.

Women will be asked standardized and sex-specific questions on access and use of services, attitudes, experiences and reasons for service use/non-use of V&A and RH issues. The population based study will measure differences in characteristics, behavior and attitudes of respondents from communities near to voucher facilities *vs*. respondents from communities far from voucher facilities but otherwise similar, as well as offer insights into preferences for the accredited services and reasons for use/non-use of these. Some of the questions may have the potential to cause distress to the women. Therefore, the research assistants will be carefully selected and will work under the supervision and support from the study coordinator. This will also provide an opportunity to validate several findings with the findings of exit client and CPI.

Provisions will be made to train researchers to ensure that guidance on ethical conduct is clearly understood and implemented. Training of research assistants is likely to take a minimum of eight days including a pretest in the field. Table 3 describes examples of operational results and indicators to compare voucher and non-voucher health facilities and communities.

**Table 3:** Examples of operational results and indicators to be used to compare results from the V&A and non V&A health facilities and communities

Objective 1: To evaluate the impact of the V&A approach on improving reproductive health behaviors and status and reducing inequities at the population level		
**Results**	**Indicators**	**Data source**

Increase in clients using maternal health care services including poor women	Clients received ANC services from public health facility Clients received institutional safe delivery care (normal/vacuum/forceps/caesarean section) Clients received pregnancy related complications management from the designated facility Clients utilized PNC services Clients received facility-based care for managing life threatening complications Clients received key physical and laboratory examinations (weight, height, blood test, urine test, abdomen exam, sonogram or ultrasound, and anemia exam).	Service statistics Population survey

Improved attitudes of service providers towards poor women	Providers indicating non discriminatory attitudes Clients recommending services to others	Client exit interview CPI Population survey

Improved quality of services	Service waiting hours Round-the-clock service availability Maintaining privacy and confidentiality Availability of necessary service equipments and logistics	Client provider interaction Client exit interview Facility inventory

Reduced out-of-pocket expenses	Medicine cost Transport cost Unofficial charge by the providers	Population survey

Reduced disease burden	Proportion of untreated complicated pregnancies	Population survey

**Objective 2: To assess the effect of the V&A approach on increasing access to, quality of, and reducing inequities in the use of selected RH services**		

**Results**	**Indicators**	**Data source**

Increased knowledge and skills of service providers on maternal health care issues	Recite proper schedule of TT and child immunization Life-threatening complications management Referral conditions for life-threatening conditions	Interviews with service providers

Increased awareness of clients on maternal health care issues among all clients and poor clients	Complications during pregnancy, delivery and post-partum period Number of ANC visits and schedule Schedule of Vitamin A capsule and iron tablet or syrup Schedule of TT and child immunization Breastfeeding	Population survey

Increased utilization of maternal health care services	Clients received ANC services from public health facility Clients received institutional safe delivery care (normal/vacuum/forceps/caesarean section) Clients received pregnancy related complications management from the designated facility Clients utilized PNC services Clients received facility-based care for managing life threatening complications Clients received key physical and laboratory examinations (weight, height, blood test, urine test, abdomen exam, sonogram or ultrasound, and anemia exam).	Population survey

Improved patient satisfaction with health care experiences	Perceived barriers to accessing services: costs, distance, quality, waiting times, privacy, confidentiality, respect, stigma surrounding service	Population survey Client exit interview

In order to enhance the findings from population surveys and address unforeseen questions arising from other components of the study, in-depth interviews (IDIs) and focus group discussions (FGDs) with healthcare providers and key informants will be conducted. These interviews will be used to gain a deeper understanding into the motivations, perceptions, and priorities of the healthcare providers regarding voucher and accreditation. The provider IDIs and FGDs will focus more specifically on: services offered; attitudes towards voucher and accreditation, including effects on workload; benefits and challenges of the voucher and accreditation; perception of clients' views; the referral system and other healthcare needs.

FGDs will be carried out with groups of male and female voucher and non-voucher users (aged 18 years and over) as well as with providers. These will take place alongside the baseline and endline surveys, as well as on an ad hoc basis when needed during the project. These will be used to gain a deeper understanding into the motivations, perceptions, and priorities of the local community regarding vouchers and service use. The FGDs will address the following broad themes: motivations for healthcare use and selection/use of the RH services; attitudes towards voucher and accreditation; communication/interaction with different providers; contraceptive and sexual health behavior, including communication with partners and other community members about RH services. All participants in the FGDs will be requested to respect confidentiality and to agree to not to divulge any information heard during the discussion outside of the group.

FGDs of one to two hours will be held in four randomly selected populations within the surveyed districts:

1 FGD: 6-8 younger women who are currently or have been voucher users (< 25 years)

1 FGD: 6-8 older women who are currently or have been voucher users (25 years & over)

1 FGD: 6-8 younger women who have never used voucher (< 25 years)

1 FGD: 6-8 older women who are have never used vouchers (25 years & over)

1 FGD: 6-8 younger men who/or partners are currently or have been voucher users (< 25 years)

1 FGD: 6-8 older men who are currently or have been voucher users (25 years & over)

1 FGD: 6-8 younger men who/or partners who have never used voucher (< 25 years)

1 FGD: 6-8 older men who/partners who have never used vouchers (> 25 years)

### Data Management and Analysis

The Data Management Unit in Population Council will store all data in password-protected computers. Hard copies of questionnaires, anonymised transcriptions and tapes of the group discussions will be stored securely in a locked cabinet, in accordance with the Population Council policy and Cambodian's data protection policy.

Analyses of facility data will be undertaken and the proportion of women receiving an acceptable quality of service will be calculated using a procedure, as described earlier, similar to Lot Quality Assurance Sampling (LQAS).

In addition, time series analyses will be conducted in order to estimate: mean monthly number of clients obtaining RH services, by type, at accredited and non-accredited providers; mean monthly number and proportion of clients in the lowest economic quintile obtaining RH services, by type, at accredited and non-accredited facilities and proportion of voucher services among all RH services at accredited facilities.

Population-level surveys provide the opportunity to measure reproductive health indicators, including both reported health status, behaviors and healthcare utilization, among populations being served by a voucher program and comparable populations not served by the voucher program. Statistical comparisons between these indicators can then be used to detect any differences between the populations at 1% and 5% level of significance. We will also compare concentration index scores for selected RH indicators calculated from the data collected among accredited and non-accredited populations. The concentration index is a widely used indicator for quantifying the degree of income related inequality in a specific health indicator and will be used to provide evidence of the extent to which voucher and accreditation approach reduces inequities. Paper questionnaires will be used to capture quantitative data. Data from paper questionnaires will be keyed into Epidata 3.1/Access and exported into Stata 10/SPSS 14 for analysis. Qualitative data will be captured on paper and audio tapes and later transcribed into MS Word before exporting into QSR Nvivo 8 for analysis using thematic framework.

## Discussion

### Ethical Issues

Informed consent will be obtained separately for each interview. For all the tools, provisions will be made to train researchers to ensure that guidance on ethical conduct is clearly understood and implemented. Such training will include sessions and exercises regarding the meaning and process of informed consent, the importance of protecting the privacy of subjects, and confidentiality of the information obtained from them. The research team will also be trained to listen and observe intently without displaying any judgmental attitude towards information they receive from the informants and on other critical ethical issues. The research teams will discuss and develop measures in relation to data recording style, personal identifiers, transcription and processing procedures, lifespan of unprocessed data, type and places of storage, and data safety and right of access.

All interviews will only be recorded after obtaining written informed consent from the interviewee. From the outset, it will be made clear to participants that they have a right to withdraw from the research at any time. At the end of the interview, participants will be provided with any necessary information to complete their understanding of the nature of the research. The researcher will discuss with the participants their experience with the research in order to monitor any unforeseen negative effects or misconceptions.

No immediate tangible benefit is likely to accrue to the subjects through their participation and this will be made clear when obtaining informed consent. However, the potential benefits to healthcare services and the women who use them will be described to potential participants, so that they are fully aware that the data gathered will be used to provide recommendations to the Cambodian Ministries of Health, as well as to healthcare providers and communities in other countries. If any informant reports problems that will require medical and or psychosocial attention, they will be referred to existing services.

If the researchers find out information or observe activities during site visits that reflect poorly on the quality of health services provided or the health system in general, this will be recorded and incorporated into a summary report. The intervention team will try to address these issues (e.g. training of healthcare providers will include interpersonal skills as well as clinical skills). If, during a client/provider interaction, a client is perceived to be at medical risk, the observer (who will be medically trained) will be trained to intervene. Given the sensitive nature of the information to be gathered, protecting and respecting the confidentiality and privacy of informants will be a critical consideration throughout the study.

All participants will receive the following information:

• Aim of the study and methods to be used

• Institutional affiliations of the research

• Anticipated benefits and potential risks and follow-up of the study

• Discomfort it may cause

• Sensitive questions regarding sexual behavior, partners and condom use will be asked, though they may choose not to answer any questions

• Questionnaire administration will increase time at clinic

• Right to abstain from participating in the study, or to withdraw from it at any time, without reprisal

• Measures to ensure confidentiality of information provided

• Study numbers will be used on questionnaires to maintain anonymity of study participants

• No information will be divulged to partners or other third parties

• Monetary compensation will only be provided if participant has to travel for the interview

• Contact details of the study coordinator for any questions or concerns

During health facility assessments, informed consent will be obtained jointly for the observation and exit interview. During the recruitment interviews at the facilities, women will be asked a number of potentially sensitive questions, including their reproductive behavior and perceptions of contraceptive use. To avoid the risk of others overhearing this information, interviews will be conducted in strictly private settings with and ample time to ensure that privacy and confidentiality can be guaranteed.

During the provider interviews, respondents will be assured that no one, including their supervisor, will know what they say and their names will not appear on the questionnaire. However, anonymised summary information and opinions from providers will be presented as part of the summary report. Results of client and provider interviews will be presented in reports in an aggregated manner such that responses cannot be traced back to individuals. Population Council staff will visit the data collection site to ensure interviewers adhere to confidentiality procedures.

During the population survey, written consent will be administered to all respondents. According to Cambodian law, the age of consent for participating in research is 18 years. Given that it would be difficult to trace parents of adolescents attending FP clinics no one will be interviewed under 18 years of age. However for the community survey any adolescents aged 15-17 years will be interviewed at the household level only if an adolescent assents and parental consent has been obtained first.

Names will only be recorded for women agreeing to any follow-up visits to facilitate contact. This information, together with their contact information, will be recorded on a separate sheet and kept physically separate from the data collection instruments containing their information, and will only be linked to questionnaires by study numbers. Only the research team coordinator will have access to both the names and instruments. During follow-up interviews, a woman may not want others to know that she is participating in the study, and all women will be offered the opportunity to hold the interview in a location of her choosing, with travel costs reimbursed to ensure that this is a viable option. The majority of clients will be interviewed at the clinic or during community surveys. However if/where the informants are requested to travel specifically for interview, the study will compensate informants with a small gift, typically a Cambodian scarf, for any inconvenience. Researchers conducting FGDs will be reminded how to preserve confidentiality and ask the groups to respect personal information before discussions start.

### Ethical Clearance

This research protocol has been reviewed by key stakeholders and ethical clearance has been granted by National Institute of Public Health (NIPH) Cambodia and the Population Council Institutional Review Boards.

## List of abbreviations

ANC: Antenatal Care; BMGF: Bill and Melinda Gates Foundation; CPI: Client Provider Interaction; EPOS: German consulting firm serving as lead partner in voucher management; FP: Family Planning; GOC: Government of Cambodia; GTZ: German Technical Corporation; HFA: Health Facility Assessment; IDI: In-depth Interview; IRB: Institutional Review Board; LAM: Lactational Amenorrhea Method; LQA: Lots Quality Assurance; MCH: Maternal and Child Health; MDG: Millennium Development Goal; MIS: Management Information System; MNH: Maternal and Newborn Health; MOH: Ministry of Health; NGO: Non Government Organization; OBA: Output-Based Aid; OD: Operational District; PDA: Personal Digital Assistant; PNC: Postnatal Care; PPS: Probability Proportional to Size; RH: Reproductive Health; SPSS: Statistical Package for Social Science; STI: Sexually Transmitted Infection; SRH: sexual and reproductive health; WHO: world health organization.

## Competing interests

The authors declare that they have no competing interests.

## Authors' contributions

BB and CW were involved in the conceptual design of the study. BB and CW were involved in drafting, re-organizing and overall revision of the manuscript. TR, SV, CC, HS, CM, AB and UR were involved in the revision of the manuscript. All the authors have read and approved the final manuscript.

## Pre-publication history

The pre-publication history for this paper can be accessed here:

http://www.biomedcentral.com/1471-2458/11/667/prepub
